# Takotsubo cardiomyopathy complicated by persistent complete heart block treated with conduction system pacing: A case report

**DOI:** 10.1016/j.ipej.2026.07.001

**Published:** 2026-07-03

**Authors:** Ohanian Antranik, Nahouli Lara, DA Sami Augustin

**Affiliations:** a Cardiac Electrophysiology Fellow, Centre Hospitalier Moulins-Yzeure, France; b Obstetrics and Gynecology Department, American University of Beirut Medical Center, Lebanon; c Cardiology Department, Centre Hospitalier Emile Roux, France

## Abstract

Takotsubo cardiomyopathy is a transient form of stress-induced cardiomyopathy triggered by emotional or physical stressors. Although cardiac complications occur in nearly half of patients, atrioventricular conduction disturbances are rare, and the optimal management strategy, including the decision regarding pacemaker implantation and its timing, remains uncertain. We report the case of an 80-year-old woman who presented with Takotsubo cardiomyopathy and persistent complete heart block requiring pacemaker implantation with conduction system pacing despite recovery of the left ventricular systolic function.

## 1 Introduction

Takotsubo syndrome (TS) is a reversible, non-ischemic cardiomyopathy characterized by transient left ventricular wall motion abnormalities, chest pain, electrocardiographic changes and mild elevation of cardiac biomarkers in the absence of coronary artery disease [1]. It has been reported to have a prognosis like that of acute coronary syndrome [2], with comparable early and late mortality [3]. The estimated annual incidence is approximately 1-2% among patients undergoing angiography for suspected acute coronary syndrome [3]. Cardiac complications occur in nearly half of patients having TS, and in-hospital mortality is estimated at 4% to 5%, mainly due to cardiogenic shock, mechanical complications and life-threatening arrhythmias [3].

Complete atrioventricular (AV) block in patients presenting with TS is very rare [4], with a reported incidence of 2.9% [5]. Moreover, conduction system pacing has not been well studied in patients with Takotsubo cardiomyopathy.

Although the optimal management of patients with TS presenting atrioventricular conduction disease remains uncertain, and despite the reported cases of AV block resolution after recovery of left ventricular wall motion abnormalities [6], evidence suggests that AV block often persists after left ventricular systolic function recovery [1].

We report the case of an elderly woman who presented with TS and complete AV block that persisted for several days after recovery of left ventricular ejection fraction (LVEF), requiring pacemaker implantation. We discuss the possible causal relationship between TS and AV conduction disturbances, as well as the management of this complication, including the indication and timing of pacemaker implantation and the pacing modality according to the best available evidence.

## 2 Case presentation

An 80-year-old woman with a history of hypertension was admitted to another hospital with chest pain and a one-week history of dyspnea that began after a significant emotional stressor, namely an attempted armed burglary at her home.

On presentation, she showed signs of left-sided heart failure. The electrocardiogram (ECG) showed regular sinus rhythm with complete AV block, narrow-complex escape rhythm, mild diffuse ST-segment elevation, and a long QT interval (Fig. 1A). No pre-event ECG was available for review, as the patient was not previously followed at the institution and first presented with complete AV block.

**Fig. 1:**
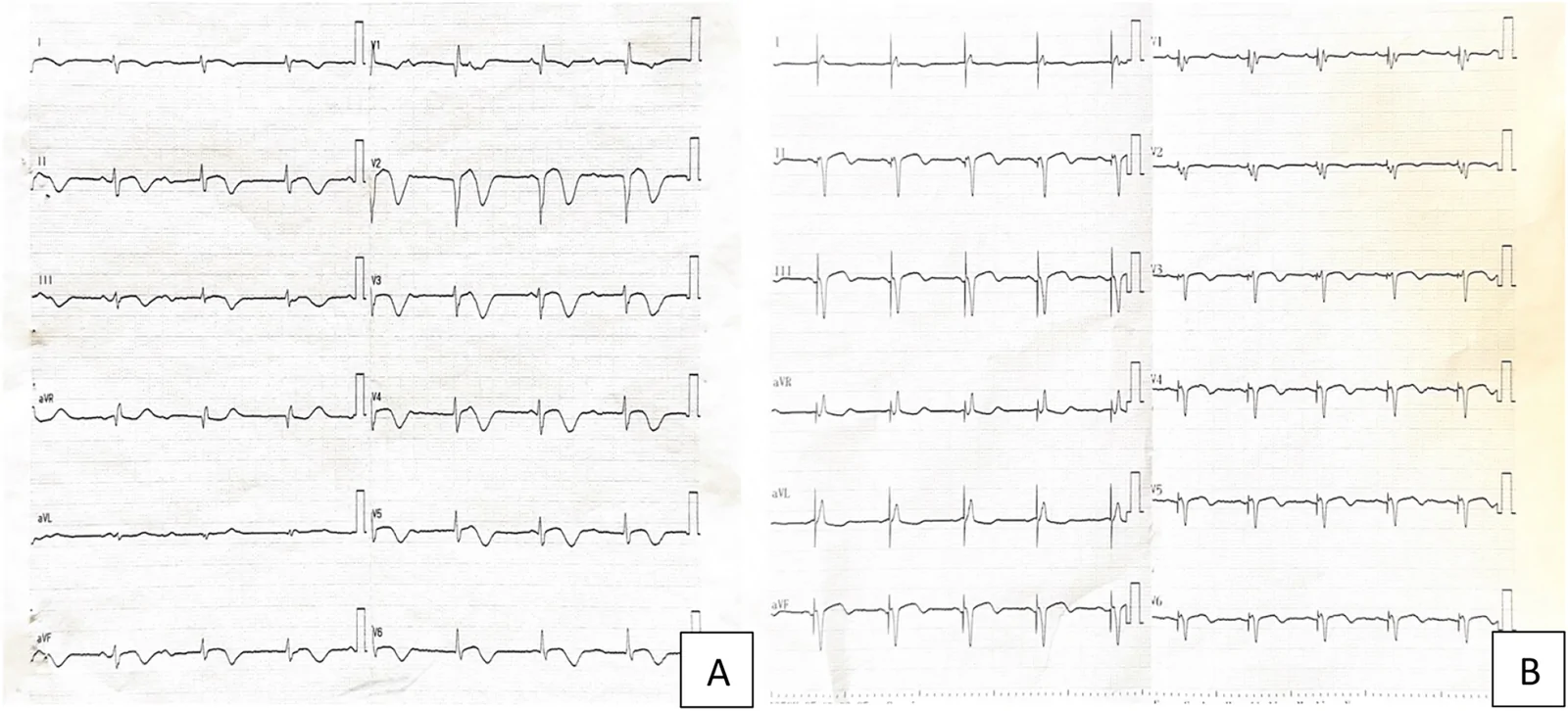
A: 12-lead ECG on admission showing complete AV block. B: 12-lead ECG after dual-chamber pacemaker implantation with LBBAP, showing capture of the posterior fascicle.

Transthoracic echocardiogram showed moderate LV systolic dysfunction with an LVEF of 35–40% and apical akinesia (Fig. 2). Troponin was mildly elevated at 142 pg/mL. Because of chest pain, electrocardiographic changes, apical akinesia on transthoracic echocardiography, and mild troponin elevation, acute coronary syndrome was initially suspected, and the patient received a loading dose of aspirin and parenteral anticoagulation. However, coronary angiography showed normal coronary arteries (Fig. 4), and left ventriculography demonstrated typical features of Takotsubo syndrome, with apical ballooning and preserved contraction of the basal segments (Fig. 3). Antithrombotic therapy was therefore discontinued. The patient was managed with continuous telemetry monitoring and supportive heart failure therapy mainly with an angiotensin-converting enzyme inhibitor, aldosterone antagonist and SGLT2-inhibitor. Electrolytes were monitored and corrected, QT-prolonging medications were avoided, and beta-blocker therapy was not initiated because of persistent complete AV block.

**Fig. 2:**
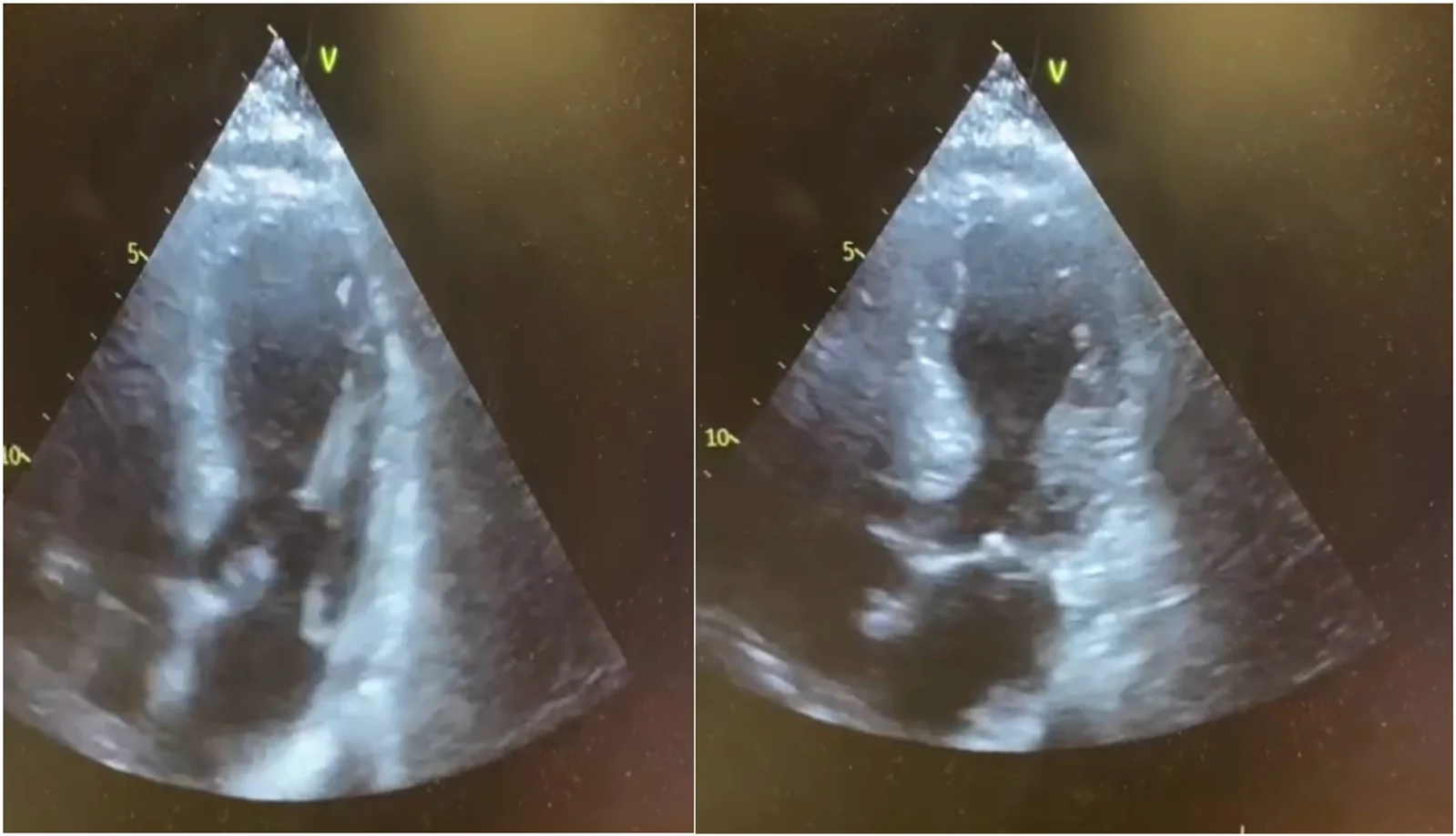
Transthoracic echocardiography showing apical akinesia in systole.

**Fig. 3:**
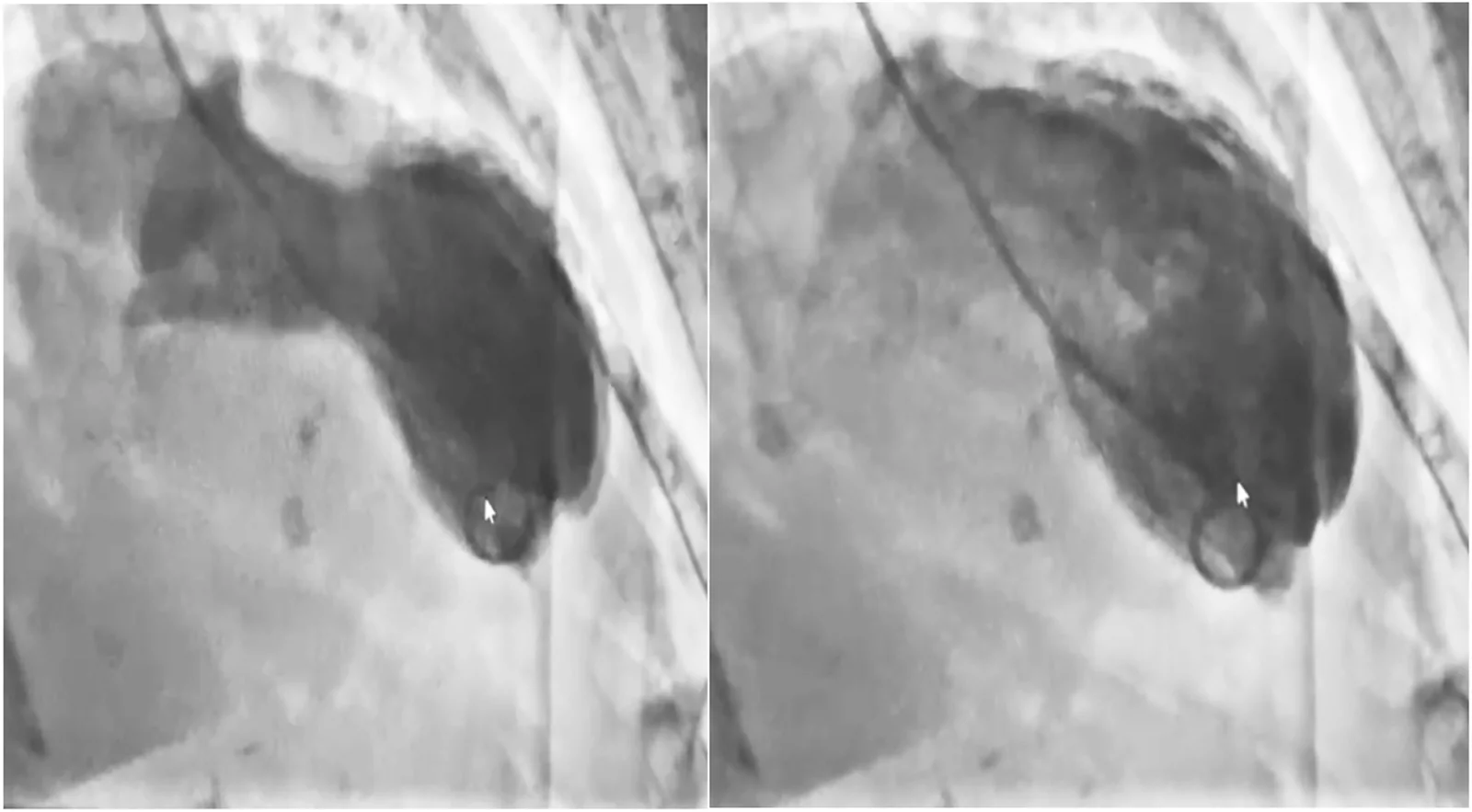
Left ventriculography showing apical ballooning.

**Fig. 4:**
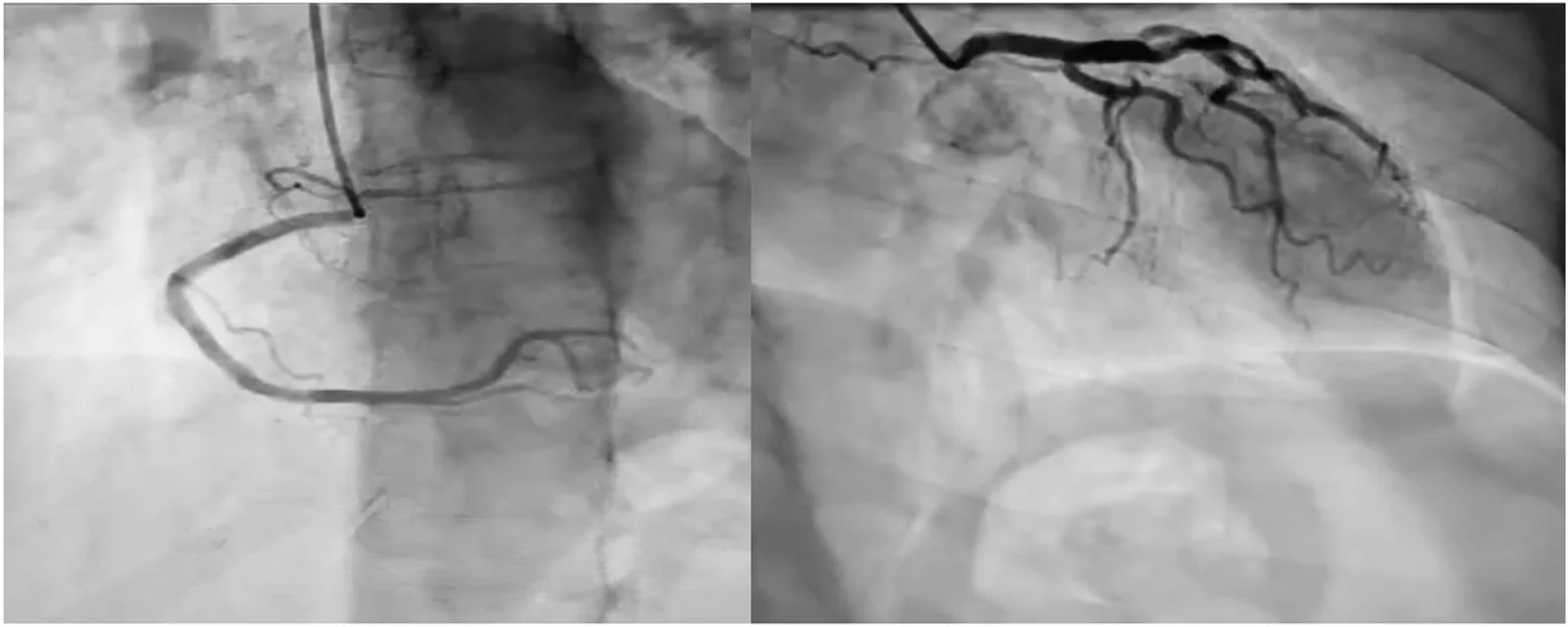
Coronary angiography showing normal coronary arteries.

On day 5, repeat transthoracic echocardiography showed improvement in left ventricular systolic function, with an LVEF of 50%. Given the clear emotional stressor, typical findings on echocardiography (Fig. 2) and left ventriculography (Fig. 3), absence of obstructive coronary lesions (Fig. 4), and early improvement in left ventricular systolic function, the diagnosis of Takotsubo syndrome was established. Cardiac magnetic resonance imaging (CMR) was not performed during the index hospitalization because the diagnosis was considered sufficiently supported by the clinical context, typical apical ballooning, normal coronary angiography, and early recovery of left ventricular systolic function. There were no clinical features strongly suggestive of myocarditis or infiltrative cardiomyopathy, and CMR was not expected to change the acute management. However, outpatient CMR follow-up was planned to further assess myocardial tissue characterization after clinical stabilization.

On day 7, given the persistence of complete AV block despite improvement in left ventricular systolic function, permanent pacing was favored over prolonged observation. The patient was transferred to our hospital, where dual-chamber pacemaker implantation using left bundle branch area pacing was performed. The post-implantation ECG showed capture of the posterior fascicle (Fig. 1B). The implantation was performed under local anesthesia with lidocaine and close hemodynamic monitoring. The patient remained calm throughout the procedure, and no additional sedative or anxiolytic therapy was required. Two weeks after implantation, and despite recovery of left ventricular function, AV block persisted, and the patient became pacemaker dependent.

## 3 Discussion

Stress-induced cardiomyopathy is a reversible form of cardiomyopathy first described in Japan in 1990 by Sato et al. [7] under the name of takotsubo cardiomyopathy because of its resemblance to the Japanese octopus pot [5]. It most commonly affects postmenopausal women and is triggered by physical or emotional stressors [8]. TS is characterized by transient regional wall motion abnormalities, including akinesia, dyskinesia or hypokinesia; the apical variant is the most common (81.7%) followed by mid-ventricular (14.6%), basal (2.2%) and focal (1.5%) variants [9].

Although its pathophysiology is not fully understood, proposed mechanisms include coronary artery spasm, microvascular dysfunction, dynamic mid-ventricular cavity or left outflow tract obstruction and direct toxicity of the catecholamines on cardiomyocytes [5].

TS may be associated with several life-threatening complications which include, left ventricular thrombus formation, dynamic left ventricular outflow tract (LVOT) obstruction, cardiogenic shock and cardiac arrhythmias [10]. The latter have been reported as unusual complications of TS including atrial fibrillation, ventricular arrhythmia and conduction system disease [1]. The combination of complete heart block and TS has been rarely reported in the medical literature with two studies reporting a prevalence of complete AV block of 2.2% and 2.8%, respectively representing one the rarest complications [1].

Several theories have been proposed to explain the occurrence of AV block in TS. One hypothesis is that the spasm of the microcirculation that is caused by the catecholamine release mimics ischemia or causes an increased vagal tone [10]. Another theory is that myocardial edema can cause AV dysfunction [10]. A further possibility, is that the diffuse fibrosis or micronecrosis, as identified by cardiac magnetic resonance in some patients with malignant arrhythmias, reflects an underlying degenerative or infiltrative process that contributes to conduction disease [10].

Two main questions arise in case of AV block associated with TS. First, what is the causal relationship between the two conditions? Second, is the conduction disturbance reversible, and how long should clinicians wait before pacemaker implantation?

The temporal and causal relationship between AV block and TS remains difficult to establish. Three mechanisms may be considered: AV block may act as a physical stressor capable of precipitating TS; TS may transiently impair AV conduction through autonomic imbalance, microvascular dysfunction, or myocardial edema; or both conditions may coexist, particularly in elderly patients with underlying degenerative conduction system disease [6,10]. In our patient, the clear emotional trigger, typical apical ballooning pattern, normal coronary angiography, and early recovery of left ventricular systolic function support TS as the primary diagnosis. The narrow QRS complex escape rhythm on ECG argues against overt pre-existing His–Purkinje system disease; however, in the absence of a pre-event ECG, an intermittent or paroxysmal conduction abnormality cannot be completely excluded. Moreover, the persistence of complete AV block despite recovery of left ventricular systolic function suggests that the conduction disturbance was not merely a transient consequence of acute ventricular dysfunction. Therefore, rather than assuming a single causal direction, this case is best interpreted as TS complicated by persistent complete AV block, while acknowledging that the exact sequence between the two conditions cannot be definitively established.

The management of high-grade AV block in the setting of TS remains challenging, and the appropriate timing of permanent pacemaker implantation is unclear [1]. Although transient recovery of AV conduction has been reported, most published cases describe persistent AV conduction disturbances despite recovery of left ventricular systolic function, with many patients ultimately requiring permanent pacemaker implantation [10,11]. Accordingly, when complete AV block does not resolve promptly after improvement of left ventricular function, permanent pacing may be considered without a prolonged delay [1]. In our patient, the persistence of complete AV block for one week after presentation despite improvement in LVEF supported the decision to proceed with permanent pacemaker implantation. The choice of LBBAP was based on the expectation of a high ventricular pacing burden due to persistent complete AV block. In such patients, conduction system pacing may preserve a more physiological ventricular activation pattern compared with conventional right ventricular apical pacing and may reduce the risk of pacing-induced ventricular dysfunction. The patient subsequently became pacemaker dependent.

Although pacemaker-induced TS has been reported, no studies have compared different pacing modalities, such as right ventricular apical pacing versus LBBAP, in patients with TS and AV conduction disorders. Most reported cases of pacing-induced TS have involved right ventricular apical pacing. However, rare cases of LBBAP-induced TS have also been described [12]. In our patient, LBBAP was well tolerated hemodynamically in the acute post-implantation phase, without evidence of dynamic LVOT obstruction.

This case has several limitations. First, no pre-event ECG was available; therefore, pre-existing conduction disease could not be completely excluded. Second, CMR was not performed during the index hospitalization, limiting immediate assessment for myocardial edema, fibrosis, or alternative diagnoses such as myocarditis; however, outpatient CMR follow-up was planned after clinical stabilization.

## 4 Conclusion

The management of patients presenting with TS and AV conduction disorders remains challenging, particularly regarding the indication and timing of permanent pacemaker implantation. The available evidence shows that in most patients AV conduction disturbances may persist even after LV recovery requiring pacemaker implantation. More data are needed to evaluate the best pacing strategy (RV apical vs CSP) in this population.

## Consent statement

I, the undersigned, hereby give my consent for the publication of my medical case details and any related images in a scientific journal. I understand that my name will not be published, and all efforts will be made to protect my identity.

## Ethical approval and consent

Written informed consent was obtained from the patient for publication of this case report and accompanying images. Institutional review board approval was not required for anonymized single case reports according to institutional policy.

## Authors contributions

Antranik Ohanian: patient care, conceptualization, data acquisition, electrophysiological interpretation, literature review, and manuscript drafting. Sami Augustin DA: patient care, data acquisition, clinical interpretation, and critical revision. Lara Nahouli: manuscript drafting, language editing, and manuscript revision. All authors reviewed and approved the final submitted manuscript.

## Ethical statement

Informed Consent for publication was obtained from the patient.

## Funding

No funding from an external source supported the publication of this case report.

## Declaration of competing interest

The authors declare that they have no known competing financial interests or personal relationships that could have appeared to influence the work reported in this paper.
